# Beyond 2D: effects of photobiomodulation in 3D tissue-like systems

**DOI:** 10.1117/1.JBO.25.4.048001

**Published:** 2020-04-29

**Authors:** Polina Y. Bikmulina, Nastasia V. Kosheleva, Anastasia I. Shpichka, Yuri M. Efremov, Vladimir I. Yusupov, Peter S. Timashev, Yury A. Rochev

**Affiliations:** a Sechenov First Moscow State Medical University, Institute for Regenerative Medicine, Moscow, Russia; b Lomonosov Moscow State University, Faculty of Biology, Moscow, Russia; c FSBSI “Institute of General Pathology and Pathophysiology,” Moscow, Russia; d FSBEI FPE “Russian Medical Academy of Continuous Professional Education,” Ministry of Healthcare of Russia, Moscow, Russia; e Lomonosov Moscow State University, Chemistry Department, Moscow, Russia; f Institute of Photon Technologies of FSRC “Crystallography and Photonics” RAS, Troitsk, Moscow, Russia; g N.N. Semenov Institute of Chemical Physics, Department of Polymers and Composites, Moscow, Russia; h National University of Ireland, National Centre for Biomedical Engineering Science, Galway, Ireland

**Keywords:** photobiomodulation, tissue engineering, hydrogel, fibrin, mesenchymal stromal cells, regenerative medicine

## Abstract

**Significance:** Currently, various scaffolds with immobilized cells are widely used in tissue engineering and regenerative medicine. However, the physiological activity and cell viability in such constructs might be impaired due to a lack of oxygen and nutrients. Photobiomodulation (PBM) is a promising method of preconditioning cells to increase their metabolic activity and to activate proliferation or differentiation.

**Aim:** Investigation of the potential of PBM for stimulation of cell activities in hydrogels.

**Approach:** Mesenchymal stromal cells (MSCs) isolated from human gingival mucosa were encapsulated in modified fibrin hydrogels with different thicknesses and concentrations. Constructs with cells were subjected to a single-time exposure to red (630 nm) and near-infrared (IR) (840 nm) low-intensity irradiation. After 3 days of cultivation, the viability and physiological activity of the cells were analyzed using confocal microscopy and a set of classical tests for cytotoxicity.

**Results:** The cell viability in fibrin hydrogels depended both on the thickness of the hydrogels and the concentration of gel-forming proteins. The PBM was able to improve cell viability in hydrogels. The most pronounced effect was achieved with near-IR irradiation at the 840-nm wavelength.

**Conclusions:** PBM using near-IR light can be applied for stimulation of MSCs metabolism and proliferation in hydrogel-based constructs with thicknesses up to 3 mm.

## 1 Introduction

The formation of cell-containing structures is one of the approaches of tissue engineering in creating bioequivalent tissues and organs. Generally, three-dimensional (3D) porous materials act as a base of these structures, for example, decellularized tissues^1^^–^^3^ or hydrogels.^4^^–^^6^ Such systems can maintain cell viability, proliferative and physiological activity, and in some cases, initiate and direct cell differentiation.^7^^,^^8^ At the same time, one of the key challenges of tissue engineering is the preservation of viable cells in these constructs and transplantation of large grafts to replace extensive defects. The diffusion of oxygen and nutrients in tissues and tissue-engineering constructs is limited by distances of 100 to 200  μm.^9^^,^^10^ At larger distances, foci of necrosis often occur, and the density of living cells decreases significantly.^11^^,^^12^ Vascularization of the grafts after transplantation due to the growth of the recipient’s capillaries is a slow process; therefore, graft rejection frequently occurs due to insufficient vascularization and oxygen starvation.^6^ Thus, the lack of vascularization is one of the most common causes of a rejection of the transplants of pancreatic islets.^13^^–^^15^

Traditionally, diffusion limitations are overcome by perfusing the tissue-engineering constructs during the formation stage. The perfusion due to diffusion and convection helps substantially in the fight against hypoxia.^16^ Also, the composition of the scaffolds themselves was modified by adding components of the extracellular matrix, growth factors, and hormones that promote angiogenesis and vascularization.^7^^,^^17^ The cultivation of cells under conditions of low oxygen tension before their encapsulation in scaffolds was also suggested to increase their resistance to hypoxia afterward.^18^^,^^19^

Another approach to improve cell viability in 3D scaffolds, as well as to control the proliferation and differentiation of stem cells, is photobiomodulation (PBM).^20^ This method is based on short-term exposure to low-intensity monochromatic (laser) or nonmonochromatic (LED) light in the visible and near-infrared (IR) spectral regions.^21^^–^^24^ The PBM effects depend on the type of cells, their state, as well as on the wavelength, dose, and intensity of the irradiation.^23^^–^^28^ Various studies had shown that light irradiation can act as an agent that directs the differentiation of cells, stimulates their survival, and activates metabolism. For example, the red 630-nm LED irradiation with an energy dose of $2.5\cdot 10^{5}\;J/m^{2}$ increased the viability of odontoblast-like cells isolated from tooth pulp.^29^ Irradiation with the near-IR 840-nm light with an energy dose of $4\;J/{\mathrm{cm}}^{2}$ stimulated the synthesis of type I collagen, and, with an energy dose of $25\;J/{\mathrm{cm}}^{2}$, it improved the adhesion of neural stem cells. Moreover, the proliferation of neural stem cells immobilized in a gelatin-methacrylate matrix increased by 44% after exposure with low-intensity laser irradiation at 635-nm wavelength and an energy dose of $62.5\;J/{\mathrm{cm}}^{2}$.^30^ The authors of this study had also provided the data suggesting that such an effect can enhance cell differentiation in the late stages of cultivation. In another study,^31^ it was shown that tooth pulp stem cells immobilized in bioceramics better differentiate in the osteogenic direction after low-intensity laser irradiation (the 660-nm wavelength and a dose of 2 to $4\;J/{\mathrm{cm}}^{2}$). Also, irradiation with a wavelength of 780 nm effectively stimulated osteogenic differentiation of cells and osseointegration in titanium scaffolds *in vivo* in a model of osteoporosis.^32^ Near-IR irradiation accelerates a new bone formation and osseointegration of transplanted cells in bone defects in the calvaria of rabbits.^33^ And while the mechanisms of the effect of red and IR irradiation on the cell are mostly similar,^34^ IR irradiation is considered more promising for 3D structures due to its ability to penetrate deep into tissues.^35^^,^^36^ Overall, light in the red and near-IR ranges with fluences around $3\;J/{\mathrm{cm}}^{2}$ was found to be the most beneficial for 3D systems.^33^^,^^37^^–^^39^

The main advantages of PBM are ease of use and noninvasiveness. It is suggested that the PBM can be used as a method of cell preconditioning before transferring cells in adverse conditions.^40^ However, the mechanisms and effects of irradiation on cells were studied mainly on monolayer cultures; there is almost no information on the effects of the PBM in 3D tissue-engineering structures. Optically transparent hydrogels are well suited to establish the effects of the PBM in 3D systems. Thus, a 3-mm-thick hydrogel layer of poly (N-vinylpyrrolidone), polyethylene glycol (PEG), agar, and water is 92% and 98% transparent for irradiation at the wavelengths of 660 and 808 nm, respectively.^41^

Mesenchymal stromal cells (MSCs) are on top of clinical interest because of their potential use in autologous transplantation. Currently, more than 2000 patients received autologous or culture-expanded allogeneic MSCs for the treatment of different diseases.^42^ Gingival mucosa is one of the promising sources of MSCs due to availability and the minimal invasiveness of its procurement as well as the ability of gingival mucosa wounds to heal without formation of scar.^43^^,^^44^ Gingival tissue, as it is routinely discarded after resective periodontal surgery, is ideal for the isolation of the human MSCs during routine procedures under local anesthesia.^45^ Human gingiva-derived MSCs produce adhesive, homogeneous, stably well-proliferating cell populations that preserve the karyotype and have pronounced anti-inflammatory and immunomodulating activity.^46^^,^^47^ Gingiva-derived MSCs can differentiate effectively in the osteogenic^48^^,^^49^ and myogenic directions^50^^,^^51^ both in 2D monolayer cultures and in 3D culture conditions, which makes the use of gingiva-derived MSCs very promising for the formation of grafts and tissue-engineering structures for recovery of the musculoskeletal system.^7^^,^^52^ MSCs from various sources are widely used in the creation of 3D tissue-engineering constructs, in particular, in combination with fibrin hydrogel, for creating a bioequivalent of the skin, cartilage, and blood vessels.^53^^–^^55^ Therefore, the investigation of the effects of the PBM on such tissue-engineering constructions is of fundamental and applied importance.

The aim of this work was to test the possibility of using PBM in the red and near-IR wavelengths for tissue engineering and regenerative medicine on a model of the formation of hydrogel grafts with encapsulated MSCs from gingival mucosa. For this, we measured the physicochemical and mechanical characteristics of the hydrogels and evaluated the viability, proliferative, and metabolic activity of MSCs in 3D hydrogels of different thicknesses and concentrations of gel-forming proteins.

## 2 Materials and Methods

### 2.1 Hydrogel Components and Formation

Fibrin gel was prepared using fibrinogen and thrombin stock solutions. Lyophilized bovine fibrinogen (Sigma Aldrich, Germany) was dissolved with sterile phosphate-buffered saline (PBS) to a concentration of 50  mg/mL, lyophilized bovine thrombin (Sigma Aldrich)—100  U/mL. Stocks were stored at −20°C before use. The used modification of fibrinogen was previously described^7^^,^^55^^,^^56^ and performed at a day of experiment by adding 1.5  mg/mL solution of O,O′-bis[2-(N-succinimidyl-succinylamino)ethyl]polyethylene glycol (PEG-NHS; Sigma-Aldrich, Germany) in PBS at a molar ratio of 5:1 (PEG-NHS: fibrinogen). The reaction of PEGylation was carried out for 2 h at 37°C. Then, 25 or 50  mg/mL fibrinogen was mixed equally with 5  U/mL thrombin to encapsulate cells. We used three different hydrogel types varying in fibrinogen concentration and final hydrogel thickness in a well (Table 1).

**Table 1:** Different types of modified fibrin hydrogel.

Type	Thickness in a well (mm)	Fibrinogen concentration (mg/mL)
Standard	1.5	25
Thick	3	25
Concentrated	1.5	50

### 2.2 Hydrogel Characterization

#### 2.2.1 Confocal laser scanning microscopy

The procedures were performed, as described elsewhere.^57^^,^^58^ Briefly, before polymerization, fibrinogen solutions were mixed with fibrinogen conjugated with AlexaFluor-488 (Invitrogen, USA) at a ratio 50:1. Samples were prepared on slides and analyzed using a confocal laser scanning microscope LSM 880 equipped with an AiryScan module and GaAsP detector (Carl Zeiss, Germany; 40× water immersion objective).

#### 2.2.2 Atomic force microscopy

The mechanical measurements on gels were performed using an atomic force microscope Bioscope Resolve (Bruker, USA). The arrays of force–distance curves were acquired in the force volume mode with CP-PNP-BSG cantilevers (NanoandMore GmbH, Germany), which had a 5  μm borosilicate glass microsphere attached as a probe. The spring constant of the cantilever was measured by the thermal tune method (0.056  N/m). The measurements were performed in the PBS medium at a temperature of 25°C. The processing of force–distance curves were conducted using MATLAB software (MathWorks). The elastic modulus E (Pa) was extracted by fitting the extend curves with the Hertzian contact mechanic model; the standard linear solid model was used to calculate the apparent viscosity from the hold region between the extend and retract phases (stress–relaxation experiments) using a numerical algorithm proposed in Ref. 59.

#### 2.2.3 Gel spectrophotometry

To reveal the gel impact in transmission of low-intensity irradiation, we measured the absorbance spectra of the cell-free and cell-laden fibrin samples prepared in quartz cuvettes (length=10  mm) using a spectrophotometer Varian 50 Scan Cary. The cell concentration was the same as in *in vitro* cell experiments ($4.7\times 10^{5}\text{ cells}/\mathrm{mL}$). Transmission spectra were calculated as a ratio of input ($I_{0}$) and output (I) intensity.

### 2.3 Cell Culturing and Characterization

#### 2.3.1 Cell culture

Human MSCs were collected from biopsies of the gingival mucosa from the retromolar area of the healthy donors who had signed the informed consent. The cells were isolated and characterized according to the protocol described in Ref. 50. Cells were cultured in DMEM/F12 medium (1:1, Biolot, Russia) supplemented with 10% fetal calf serum (HyClone), l-glutamine (5  mg/mL, Gibco), insulin-transferrin-sodium selenite (1:100, Biolot), bFGF (20  ng/mL, ProSpec, Israel), and gentamycin (50  μg/mL, Paneco, Russia). We used MSCs of the fourth passage in this study. The cell morphology was examined using a phase-contrast microscope Primovert (Carl Zeiss).

#### 2.3.2 Immunophenotyping

To confirm that cells isolated from the gingival mucosa represented MSCs, we used a standard procedure of flow cytometry analysis in accordance with MSCs minimal criteria proposed in 2006 by the International Society for Cellular Therapy.^60^ The isolated MSCs were immunophenotyped using a microfluidic cell sorter Sony SH800 (Sony Biotechnology) with antibodies for CD44, CD90, CD105, CD73, isotype IgG, CD326, CD11b, CD45, CD14, CD34, CD31 conjugated with phycoerythrin and fluorescein isothiocyanate. The cell suspension was stained with the mixture of antibodies for 15 min in the dark (5  μL of each antibody per 1 million cells) and then loaded to the sorter. Cells of the fourth passage from six different samples (50.000 events per each) were used.

#### 2.3.3 Cell encapsulation

Cells were encapsulated within the modified fibrin gels at a concentration of $7\times 10^{4}\text{ cells}$ per well ($4.7\times 10^{5}\text{ cells }\mathrm{per}\;\mathrm{mL}$) in a 48-well plate. First, the cell suspension was added to the PEGylated fibrinogen solution, and then, it was stabilized by adding thrombin. We used three types of the fibrin gel: the standard gel with fibrinogen concentration of 25  mg/mL and thickness of 1.5 mm; the thick gel with fibrinogen concentration of 25  mg/mL and thickness of 3.0 mm; and the concentrated gel with fibrinogen concentration of 50  mg/mL and thickness of 1.5 mm. The cell morphology was examined using a phase-contrast microscope Primovert (Carl Zeiss).

#### 2.3.4 Live/dead staining

Reagent for live/dead staining (Sigma Aldrich) was prepared following the manufacturer’s instructions. After adding the reagent, the cells were incubated for 30 min in the dark at 37°C. Cell nuclei were additionally stained with Hoechst 33258 (0.04  mg/mL). Images were obtained using a laser scanning confocal microscope Olympus Fluoview FV10I (Olympus, Japan).

#### 2.3.5 AlamarBlue assay

Cell viability was assessed with AlamarBlue™ cell viability reagent (Invitrogen) following the manufacturer’s instructions. After adding the reagent, samples were stored for 2 h in the dark at 37°C. Fluorescence intensity was detected using a spectrofluorometer Victor Nivo (PerkinElmer) at 530-nm excitation wavelength and 590-nm emission wavelength.

#### 2.3.6 PicoGreen assay

DNA quantity was measured with the Quant-iT PicoGreen kit (Invitrogen). The DNA release was achieved by cell lysis after triple freezing–unfreezing cycles (30 min each). Then, we removed fibrin fibers surrounding cells with proteinase K (0.02  mg/mL) and aliquoted 100  μL of a cell lysate to a new well plate. The same volume of PicoGreen was added to cell lysate samples, and then, they were incubated for 5 min in the dark. Fluorescence intensity was detected using a spectrofluorometer Victor Nivo (PerkinElmer) at 480-nm excitation wavelength and 520-nm emission wavelength. The DNA concentration in the samples was calculated using a standard curve.

#### 2.3.7 Mitochondria quantity analysis

To reveal the changes in mitochondria quantity, we used a high-content screening system CellInsight CX7 (ThermoFisher Scientific). Cells were stained with DAPI and MitoTracker Green FM (ThermoFisher Scientific) in accordance with the manufacturer’s instructions. Every 20 min, images of the layer that is 50  μm higher than the bottom were taken in the light field and fluorescence mode (excitation: 490 nm; emission: 516 nm). For each well, we analyzed 25 central fields with the total area $0.25\;{\mathrm{mm}}^{2}$ using SpotDetector mode and measured the average fluorescence intensity caused by MitoTracker Green FM.

#### 2.3.8 Statistical analysis

Experiments were carried out at least three times to ensure the validity of the results, and the data shown are from single experiments yielding similar results to the triplicate experiments. For any given experiment, each data point represents the mean ± standard deviation. The analysis was performed using the one-way analysis of variance. Differences were assumed to be statistically significant if the probability of chance occurrence (p-value) was <0.05.

### 2.4 Photobiomodulation

#### 2.4.1 LED experimental set-up

**Fig. 1:**
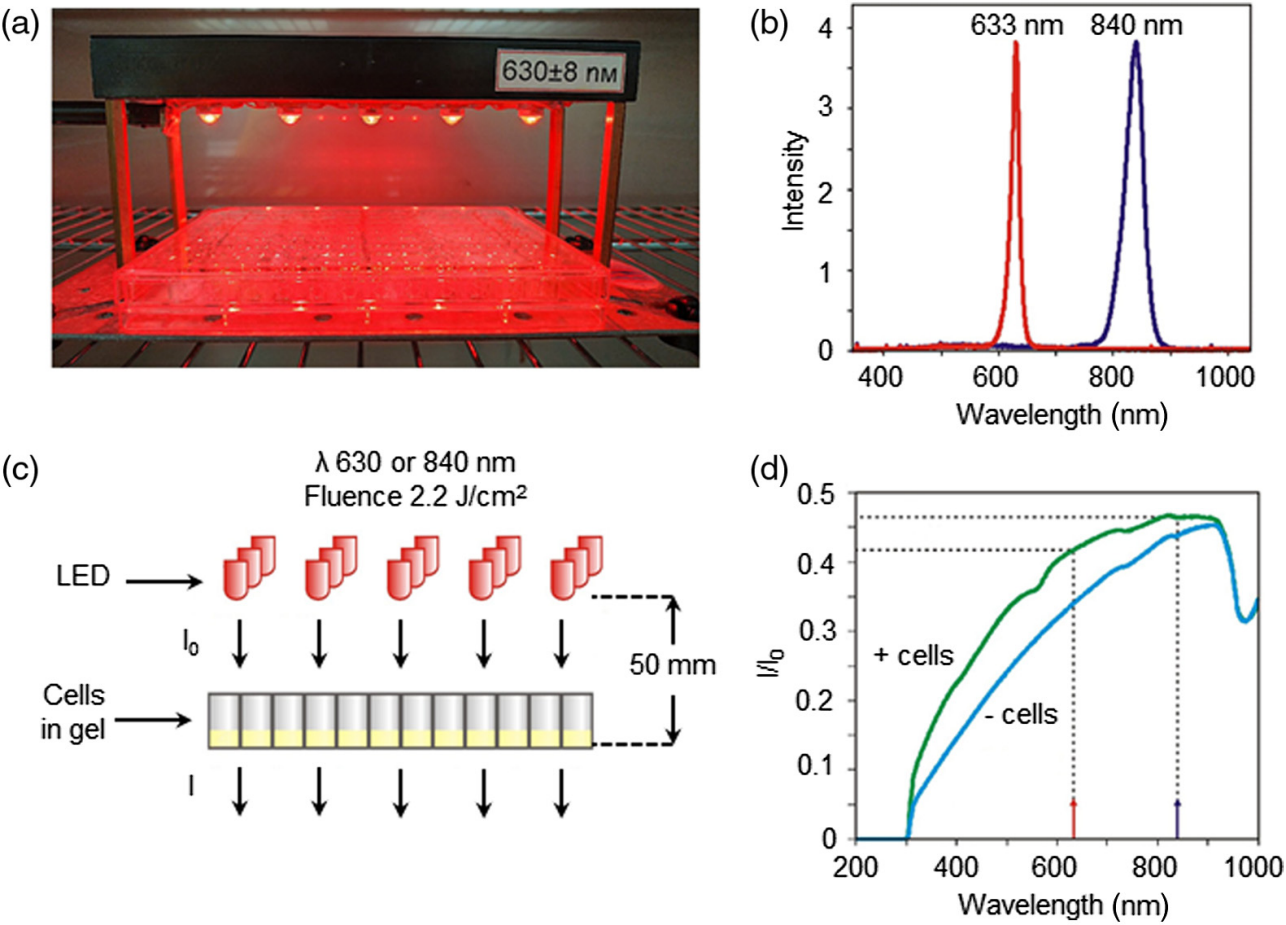
(a) The matrix irradiation apparatus LDM-07 with a plate installed for irradiation with λ=630±8  nm light. (b) Emission spectra of red and infrared irradiators normalized to their maximum intensities. (c) Scheme of the irradiation of cells in a gel with $I_{0}$ intensity. (d) Transmission spectra $I/I_{0}$ of a 1-cm-thick fibrin gel layer with and without cells.

The cells were irradiated with nonmonochromatic LED light of red [maximum at 633 nm, 16 nm full-width at half-maximum (FWHM)amplitude] and IR (maximum at 840 nm, 36 nm FWHM) ranges using the original apparatus LDM-07 with rectangular 5×11  cm LED matrices [Fig. 1(a)]. The irradiated cells were in the plate at a distance of 50 mm from the surface of the LED matrices. As a reference parameter of irradiation, we used fluence ($J/{\mathrm{cm}}^{2}$). For both wavelengths, the fluence value was the same $2.2\;J/{\mathrm{cm}}^{2}$, whereas other parameters varied due to different light sources. The characteristics of the apparatus and the exposure parameters are given in Table 2. To control the spectral composition and power of the irradiation, we used an optical fiber spectrum analyzer USB 4000 (Ocean Optics) with a wavelength range from 200 to 1100 nm and a FieldMaster power meter with a sensitive measuring head LM_10HTD (Coherent) combined with a personal computer. To reveal whether the effects of PBM were caused by light and exclude possible temperature influence, we measured a temperature using a Point Thermocouple with 0.3 mm diameter under PBM with respective parameters.

**Table 2:** Parameters of the treatment with the LDM-07 apparatus.

Light sources	Matrix of LEDs	Matrix of LEDs
Wavelength (nm)	633±8	840±18
Power (mW)	160±20	320±40
Power density ($\mathrm{mW}/{\mathrm{cm}}^{2}$)	1.8±0.2	3.6±0.4
Fluence ($J/{\mathrm{cm}}^{2}$)	2.2±0.2	2.2±0.2
Energy (J)	96±10	96±10
Time (s)	1200	600
Number of sessions	1	1

#### 2.4.2 Irradiation of cell-containing gels

The cells were irradiated in two modes a day after they were encapsulated in a hydrogel. Irradiation was conducted in the dark at a temperature of 37°C for 1200 and 600 s for red and near-IR light, respectively.

The irradiation of the plates was performed in two modes at the wavelength of 633 nm for 1200 s or at the wavelength of 840 nm for 600 s, with an energy dose of $2.2\pm 0.2\;J/{\mathrm{cm}}^{2}$ in both cases. A day after irradiation, the cell viability, proliferation, and mitochondrial activity were analyzed by a set of methods (PicoGreen assay, AlamarBlue assay, live/dead assay, and mitochondrial assay).

## 3 Results

**Fig. 2:**
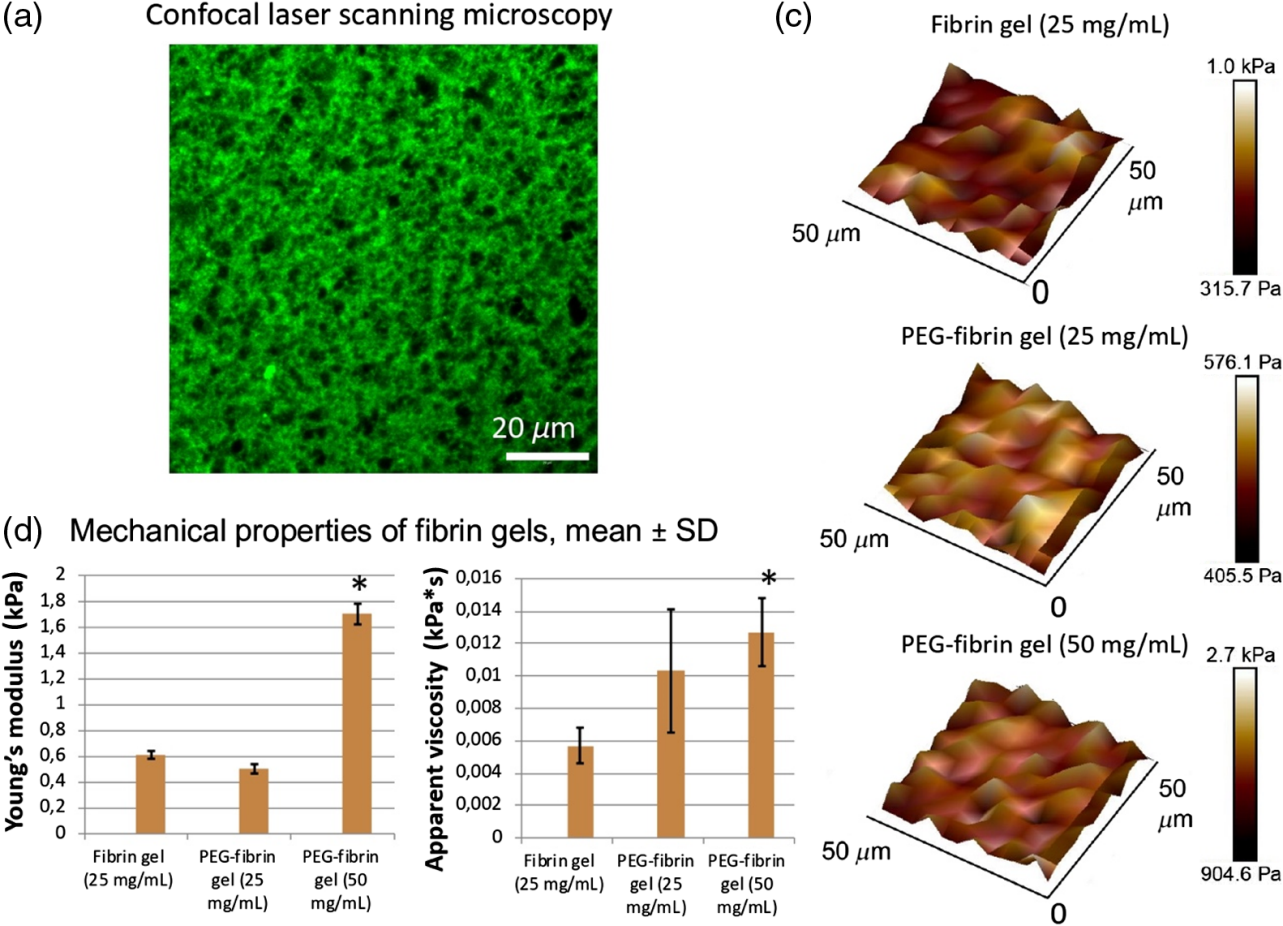
Fibrin gel characterization: (a) 5:1 PEGylated fibrin. Gel has a slightly inhomogeneous porous structure formed by short fibers; (b) mechanical properties of fibrin gels (mean±SD) measured by atomic force microscopy; (c) 3D representation of Young’s modulus distributions over a $50\times 50\;{\mu m}^{2}$ area mapped using the force volume mode. All gels demonstrated approximately the same level of the local heterogeneity of Young’s modulus.

Despite the turbidity of the native fibrin, samples of 5:1 PEGylated fibrin were transparent. The light transmission through the modified fibrin gel was high: 96% at a wavelength of 630 nm and 99% at 840 nm [Fig. 1(d)]. Interestingly, after encapsulating cells into the gel, the resulting gel transmission did not drop but actually increased [Fig. 1(d)]. Figure 2(a) shows that the PEGylated fibrin had a flocculent structure formed by short fibers; there were uniformly distributed pores varying in diameter (0.1 to 7.2  μm). All the gels measured were soft (the Young’s moduli <2  kPa), the average Young’s modulus values are presented in Fig. 2(b). The PEGylation led to a slight decrease of the Young’s modulus for the PEGylated fibrin gel (by 18%), whereas increase in fibrinogen concentration (from 25 to 50  mg/mL) led to a very pronounced increase in stiffness (by 240%) [Fig. 2(c)]. The apparent viscosity of the PEGylated gels was about twice that of the native gel.

The immunophenotype of the primary culture of MSCs obtained from the gingiva mucosa met the criteria for MSCs.^60^ The cells used in the study expressed characteristic markers of MSCs (CD90, CD73, CD105, and CD44) and did not express hematopoietic and leukocyte markers (Table 3).

**Table 3:** Immunophenotype of MSCs (passage 4) from gingival mucosa.

	Cell markers	Expression (%)
Positive	CD44	98.9±1.0
	CD90	98.6±1.7
	CD105	97.3±2.9
	CD73	98.4±1.9
Negative	Isotype IgG	0.4±0.3
	CD326	0.3±0.1
	CD11b	0.1±0.1
	CD45	0.3±0.1
	CD14	0.4±0.3
	CD34	0.1±0.1
	CD31	0.2±0.1

**Fig. 3:**
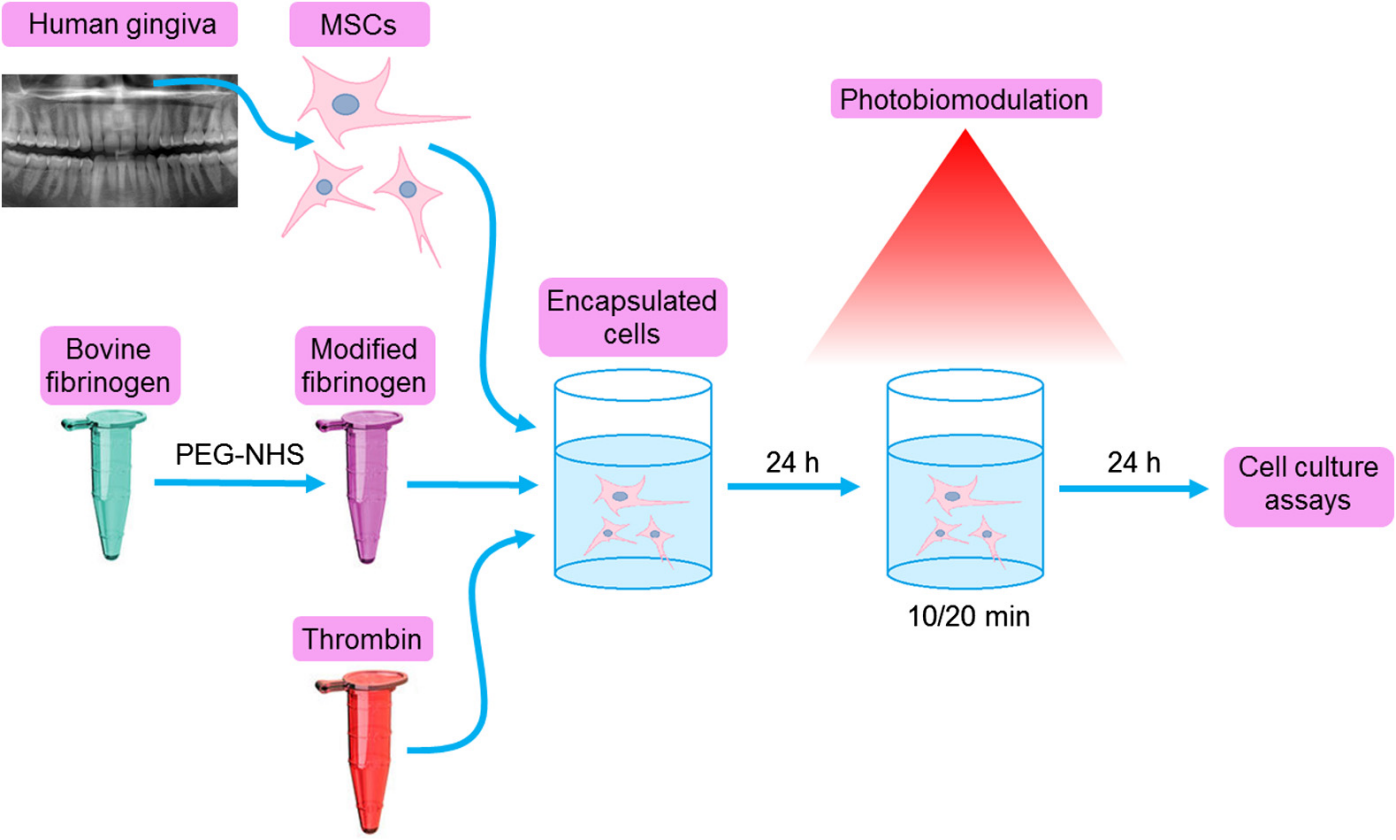
Experiment design. Mesenchymal stromal cells (MSCs) were isolated from gingival mucosa from the retromolar area and immunophenotyped. Bovine fibrinogen was modified with PEG-NHS for 2 h. To obtain a hydrogel with encapsulated cells, three components (MSCs, modified fibrinogen, and thrombin) were mixed in the wells of the plate and then incubated overnight in the dark at 37°C.

**Fig. 4:**
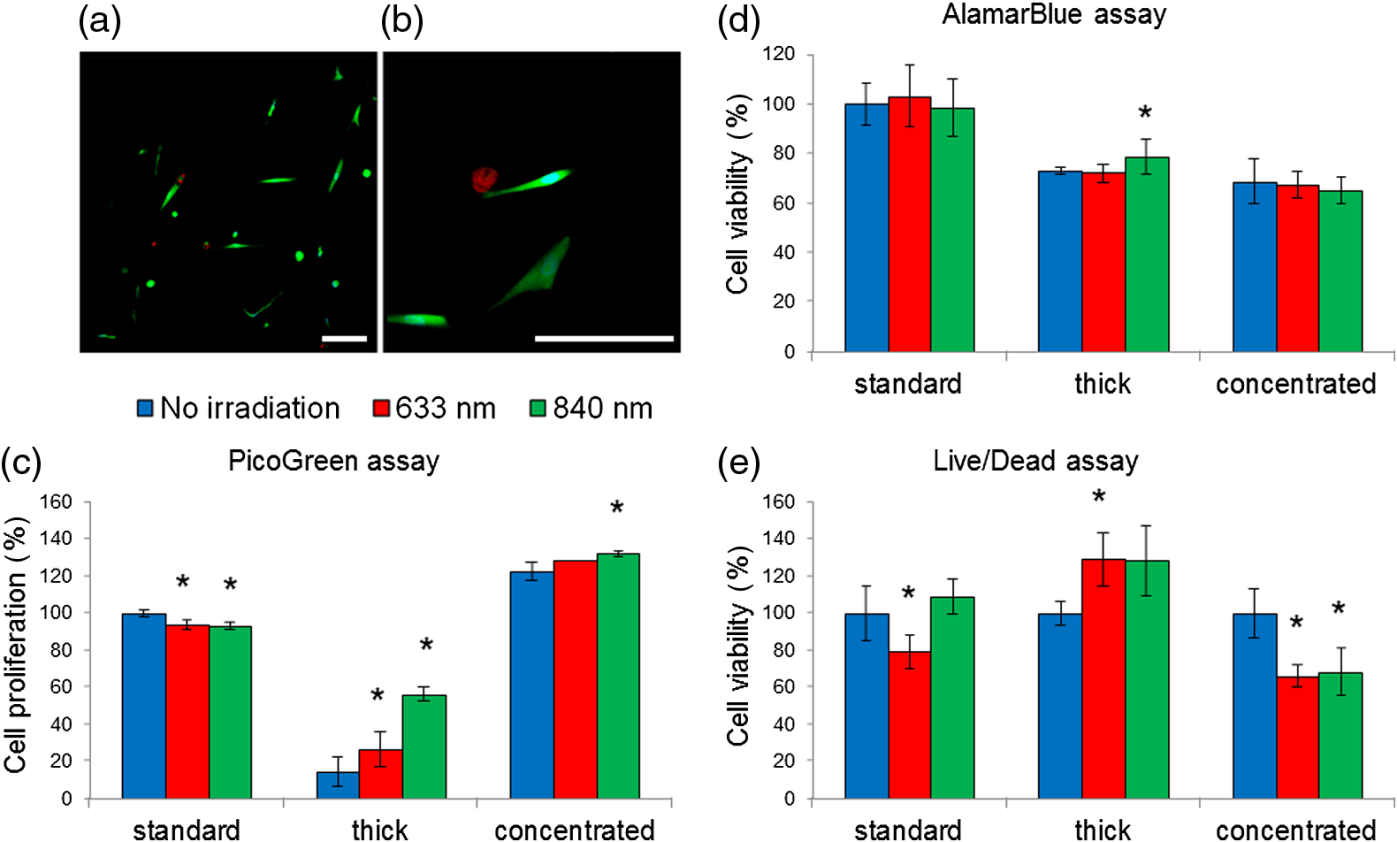
(a) Visualization of MSCs in the hydrogel (25  mg/mL) after irradiation, live/dead assay; alive cells stained with calcein (green), dead cells stained with propidium iodide (red), nuclei stained with Hoechst 33258 (blue). The scale bar is 100  μm. (b) Magnified view, the scale bar is 100  μm. (c) Results for PicoGreen assay. (d) Results for AlamarBlue assay. (e) Results for live/dead assay, calcein fluorescence intensity in living cells encapsulated in the hydrogel. Irradiation was conducted 1 day after encapsulation, and AlamarBlue, PicoGreen, and live/dead assays were conducted 1 day after irradiation. Hydrogel modifications in respect to fibrin concentration and gel thickness, respectively: standard: 25  mg/mL, 1.5 mm thick: 25  mg/mL, 3 mm; concentrated: 50  mg/mL, 1.5 mm. *p<0.05 relative to other datasets in the group.

After the encapsulation of MSCs in the gel and PBM, the effects of irradiation were analyzed by various methods for assessing cell viability and proliferation as well as by mitochondrial activity (Fig. 3). Viable cells were observed, and no significant cell death was detected in any of the samples [Figs. 4(a) and 4(b)]: the fluorescence intensity of propidium iodide was less than 1% relative to the fluorescence intensity of calcein. An assessment of the amount of DNA showed that the proliferation rate is reduced in thick gels [Fig. 4(c)], and the results of the AlamarBlue assay indicate a less active metabolism in both thick and concentrated hydrogels [Fig. 4(d)]. Thus, in hydrogels of a given thickness and concentration, the cells do not die but only switch to an inactive state, in which proliferative and metabolic activity decreases. In some cases, PBM was able to neutralize adverse conditions. The IR irradiation stimulated cell proliferation and metabolism in thick hydrogels [Figs. 4(c)–4(e)]. The results of all three tests correlated with each other: the PicoGreen assay indicated an increase in the number of cells after 840-nm irradiation by more than 2.5 times (however, the DNA content was only 60% of the control), the live/dead assay showed 20% increase, and the AlamarBlue assay showed 10% increase in metabolic activity.

The effects of PBM on cells in concentrated hydrogels manifested in a different way. The results of the AlamarBlue assay suggest that there is a tendency for a decrease of metabolic activity after irradiation [Fig. 4(d)]. Consistent with this, the data of the live/dead assay showed that the number of living cells a day after irradiation is 30% lower than that of the control [Fig. 4(e)].

**Fig. 5:**
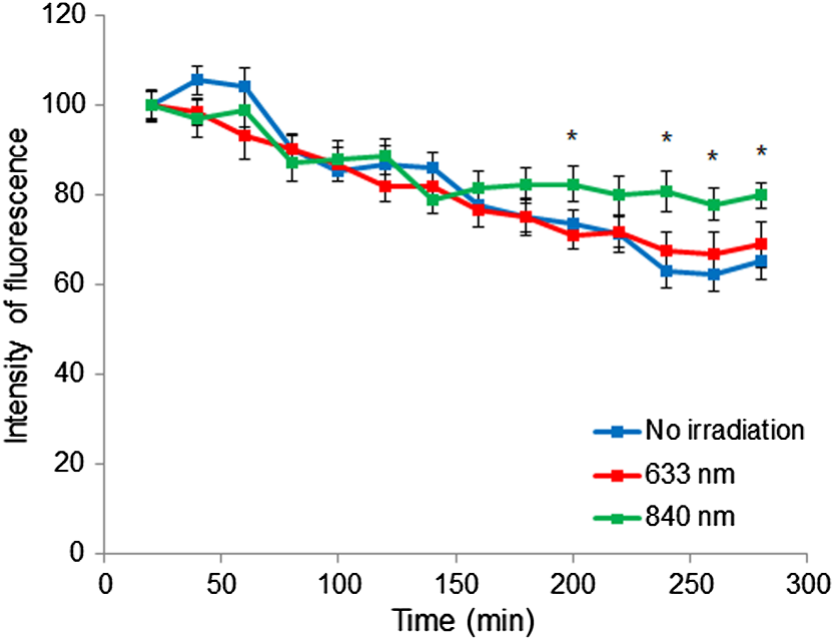
Dynamics of mitochondrial activity of the MSCs encapsulated in a hydrogel (25  mg/mL, 1.5 mm). High-content screening, MitoTracker Green. The control is the initial fluorescence intensity of MitoTracker Green for each of the datasets. *p<0.05 relative to other datasets in the group.

Tracking of mitochondria stained with MitoTracker Green, which provides an information about mitochondrial membrane potential, is widely used to understand general mitochondrial activity.^61^^–^^65^ Although the photobleaching occurs during the experiment, the relative differences still can be marked. The results of the time-lapse recording of MSCs in hydrogels showed that mitochondria of treated cells were more active and stable. The mitochondria of the encapsulated cells were also most supported by PBM with near-IR light (Fig. 5) that led to ∼23% increase in the mitochondrial activity by the end of the experiment (5 h).

## 4 Discussion

Fibrin hydrogel is a promising material for tissue engineering and regenerative medicine due to several advantages. A gel can be obtained from components of a patient’s blood; thus, it might be autologous.^66^^–^^68^ Various 3D structures can be formed from the fibrin gel, in particular, by molding and electrospinning methods.^69^^,^^70^ Moreover, the degradation rate of this hydrogel can be controlled locally, by, for example, the addition of fibrinolysis inhibitors.^71^^,^^72^ Various modifications of the hydrogel can help in directing the differentiation of cells, in particular, in the angiogenic direction.^7^^,^^55^

The data obtained on the gel structure agreed with the earlier obtained results.^5^^,^^73^ A positive correlation between fibrin gel stiffness and fibrinogen concentration was shown in previous studies that allows the preparation of fibrin gels with desired mechanical properties in a range of low Young’s modulus values (<10  kPa).^74^ The PEGylation is another factor, which affects gel stiffness by reducing it. The 5:1 PEGylated gel was also more viscous than the native one, which might be caused by smaller pore sizes. According to the poroelasticity theory,^75^ the apparent viscosity is higher due to a relaxation of a solvent moving through the porous polymer network with smaller pores.

The level of light irradiation of cells in a 3D scaffold will vary due to the absorption and scattering of light in the volume of the scaffold. Due to this variation, a part of the cells may be exposed outside the “therapeutic range” of PBM. Therefore, it is important to know the distribution of light intensity in the entire matrix, which will depend on the wavelength of the light used. This information can be provided by numerical calculation involving experimental data about effective optical materials of scaffolds. The distribution of light intensities can be estimated based on the measurement of the transmission spectra of thin films.^76^

Spectrophotometric measurements [Fig. 1(d)] showed that light transmission through the gel generally decreases with decreasing wavelength. In both curves corresponding to the gel without cells and the gel with the encapsulated cells, a clearly distinct region was presented in the edge of the IR range (920 to 1000 nm) associated with a local absorption peak of water. For the present study, it is of interest how quickly the red (λ=633  nm) and IR (λ=840  nm) light decays with depth upon irradiation of cells in a gel. Figure 1(d) shows that when cells in standard concentration are added to the gel, more light passes through the cuvette (the green curve lies above the blue). Moreover, in the case of a cell-containing gel of 1-cm thickness, the intensity of red light I decreases to 42% of the initial value ($I_{0}$) and of IR light to 47% of the $I_{0}$.

The decrease in the light intensity occurs due to its absorption and scattering on microscopic inhomogeneities of the refractive index and density of the medium. According to Beer–Lambert’s Law, the intensity of light decreases exponentially with depth in the material:

$$I(\lambda )=I_{0}\cdot e^{-\mu (\lambda )\cdot d},$$ (1)

where $I_{0}$ is the incident light intensity, I(λ) is the light intensity at a depth d, and μ(λ) is the attenuation coefficient, which depends on the wavelength. From the performed measurements, the calculated values of the attenuation coefficient are $\mu (633)=1.08\;{\mathrm{cm}}^{-1}$ and $\mu (840)=0.82\;{\mathrm{cm}}^{-1}$ for a gel without cells and $\mu (633)=0.87\;{\mathrm{cm}}^{-1}$ and $\mu (840)=0.76\;{\mathrm{cm}}^{-1}$ for a gel with cells. From Eq. (1) and the calculated attenuation coefficients, it follows that for the gel thickness of 1.5 mm, the irradiation intensities in the bottom layer will be reduced to I(633)=88% and I(840)=89% of the initial value $I_{0}$. With an increase in the gel thickness to 3 mm, the intensities in the bottom layer will be I(633)=77% and I(840)=80%.

Therefore, due to the absorption and scattering of the light in the gel, the cells will be irradiated irregularly. Exposure doses will gradually decrease for the deeper cells. In the studied gels, the irregularity of irradiation of cells in the gel with a thickness of 1.5 mm is 11% and 12%, and in the layer with a thickness of 3 mm is 20% and 23% for near-IR and red light, respectively. The difference between intensities for the red (633 nm) and near-IR (840 nm) lights is small and can be neglected.

Another important point of using PBM for 3D structures is temperature. The data obtained showed that during 600 s of irradiation with a wavelength of λ=840±18  nm and an irradiation intensity of $3.6\pm 0.4\;\mathrm{mW}/{\mathrm{cm}}^{2}$, the rate of heating is rather small and does not exceed 0.14±0.02°C. At λ=633±8  nm and an irradiation intensity of $1.8\pm 0.2\;\mathrm{mW}/{\mathrm{cm}}^{2}$ for 1200 s, the degree of heating is even less than 0.09±0.02°C. Thus, no local heating of the cells was observed.

Precise dosimetry and characterization of PBM parameters are crucial for comparing results. While intensity and duration of irradiation may vary from one light source to another, fluence depends only on light energy delivered to the object and reflects both the duration of the radiation and the intensity of the source in a linear manner.^77^ Moreover, fluence is the most indicated parameter for articles on PBM research, and using fluence as the primal parameter makes it more reliable to analyze results.^37^^,^^78^ In Refs. 31, 33, 38, 39, and 79–81, fluences from 2 to $4\;J/{\mathrm{cm}}^{2}$ were shown as the most effective for stimulating cells within scaffolds. Based on these, we chose the fluence of $2.2\;J/{\mathrm{cm}}^{2}$ for both red and near-IR light to investigate further effects.

The dependences of the fluorescence intensity (modified PicoGreen method) on the thickness of the gels [Fig. 4(c)] indicate that the activity of immobilized cells decreases with an increase in the gel thickness from 1.5 to 3.0 mm. This inhibition effect can be explained by diffusion restrictions that arise with an increase in the thickness of the scaffolds under static, nonperfused conditions. These limitations can be associated with both a lack of oxygen and a lack of nutrients. A decrease in cellular activity is a negative factor in the reconstruction of tissues and organs. To solve this problem, we proposed to stimulate cells with low-intensity irradiation with wavelengths of 633 and 840 nm. A recent study showed that blue light irradiation inhibited gingiva-derived MSCs proliferation in 2D culture, as indicated by [3-(4,5-dimethylthiazol-2-yl)-2,5-diphenyltetrazolium bromide]-test (MTT), and promoted osteogenesis.^82^

According to the results of several viability tests, changes in the physiological activity of cells in the same hydrogel samples varied greatly [Figs. 4(c)–4(e)]. Such a difference in recorded changes may be related to the sensitivity and accuracy of the methods used in these conditions. All three used assays (PicoGreen, AlamarBlue, and live/dead) are designed primarily for monolayer cell cultures. In the above experiments, a fibrin hydrogel was used as a 3D medium, which is a concentrated protein solution (5%) and acts as a turbid scattering medium. Therefore, a standard set of cytotoxicity tests might require additional calibration and optimization for 3D protein environments.

In the case of “thin gels” (thickness 1.5 mm), only a slight difference was recorded between the irradiated and unirradiated samples. In the case of gels with a thickness of 3 mm, irradiation stimulated proliferation, and this effect was especially pronounced during PBM with wavelength of 840 nm. This difference is most likely due to the specific effects of irradiation on cells. The mitochondrial respiratory chain is considered as the main target of both types of irradiation in the cell.^83^ Absorption of light by cytochrome c oxidase leads to increasing of membrane potential, exceeded ATP production, and following fluxes of protons and calcium ions.^84^ Alternative PBM mechanism involves production of a small amount of reactive oxygen species (ROS).^36^ ROS can act as mediators in several cellular pathways including kinase pathways activating cell division.^85^^,^^86^ Both of these mechanisms were shown for red and near-IR light. However, preferred paths of PBM influence on a cell may vary according to the wavelength.^87^ Thus, ROS amount produced in the cells was different for red and near-IR light with equal fluencies.^36^ Near-IR light activating cell cycle represented higher rates of ROS, which could explain more pronounced proliferation after exposure to 840 nm irradiation in the current work.

Near-IR light is more promising for tissue engineering because it is located inside the optical window and can penetrate deeper into tissue-engineered structures than red light. However, the irradiation effect was not observed in the case of thin gels with a higher concentration of fibrin (50  mg/mL). Moreover, according to the results of AlamarBlue and the live/dead assays [Figs. 4(c) and 4(d)], when cells are irradiated in concentrated gels, their viability decreases. It is possible that under conditions of increased hydrogel concentration, cells may become more sensitive to stress, and thus, irradiation of the used intensities has an adverse effect.

## 5 Conclusion

Hydrogels with an increase in the thickness or density decrease cell viability and their physiological activity. We have shown that it is possible to stimulate mesenchymal stem cell proliferation and metabolic activity in fibrin hydrogel using PBM. Thus, PBM can be used in tissue engineering to control cell populations immobilized in 3D scaffolds.

## References

[1] Agarwal T. Maiti T. K. Ghosh S. K., “Decellularized caprine liver-derived biomimetic and pro-angiogenic scaffolds for liver tissue engineering,” Mater. Sci. Eng. C 98, 939–948 (2019). MSCEEE 0928-4931 10.1016/j.msec.2019.01.037 30813101

[r2] Bölükbas D. A. et al., “The preparation of decellularized mouse lung matrix scaffolds for analysis of lung regenerative cell potential,” in Mouse Cell Culture, Methods in Molecular Biology, Bertoncello I., Ed., Vol. 1940, pp. 275–295, Humana Press, New York (2019). 10.1007/978-1-4939-9086-3_20 30788833

[3] Grebenik E. A. et al., “Chemical cross-linking of xenopericardial biomeshes: a bottom-up study of structural and functional correlations,” Xenotransplantation 26, e12506 (2019). 10.1111/xen.12506 30815940

[4] Kang B. et al., “High-resolution acoustophoretic 3D cell patterning to construct functional collateral cylindroids for ischemia therapy,” Nat. Commun. 9, 5402 (2018). NCAOBW 2041-1723 10.1038/s41467-018-07823-5 30573732 PMC6302096

[r5] Shpichka A. I. et al., “Transparent PEG-fibrin gel as a flexible tool for cell encapsulation,” Sovrem. Technol. Med. 10, 64 (2018). 10.17691/stm2018.10.1.08

[6] Park H. et al., “Three-dimensional hydrogel model using adipose-derived stem cells for vocal fold augmentation,” Tissue Eng. Part A 16, 535–543 (2010).1937-3341 10.1089/ten.tea.2009.0029 19728785

[7] Gorkun A. A. et al., “Angiogenic potential of spheroids from umbilical cord and adipose-derived multipotent mesenchymal stromal cells within fibrin gel,” Biomed. Mater. 13, 044108 (2018). 10.1088/1748-605X/aac22d 29722292

[8] Kuznetsova D. et al., “Surface micromorphology of cross-linked tetrafunctional polylactide scaffolds inducing vessel growth and bone formation,” Biofabrication 9, 025009 (2017). 10.1088/1758-5090/aa6725 28300041

[9] Radisic M. et al., “Mathematical model of oxygen distribution in engineered cardiac tissue with parallel channel array perfused with culture medium containing oxygen carriers,” Am. J. Physiol. Circ. Physiol. 288, H1278–H1289 (2005). 10.1152/ajpheart.00787.2004 15539422

[10] Riesle J. et al., “Oxygen gradients in tissue-engineered PEGT/PBT cartilaginous constructs: measurement and modeling,” Biotechnol. Bioeng. 86, 9–18 (2004). BIBIAU 0006-3592 10.1002/bit.20038 15007836

[11] Smith M. K. Mooney D. J., “Hypoxia leads to necrotic hepatocyte death,” J. Biomed. Mater. Res. Part A 80A, 520–529 (2007). 10.1002/jbm.a.30930 17013858

[12] Obradovic B. et al., “Gas exchange is essential for bioreactor cultivation of tissue engineered cartilage,” Biotechnol. Bioeng. 63, 197–205 (1999). BIBIAU 0006-3592 10.1002/(SICI)1097-0290(19990420)63:2<197::AID-BIT8>3.0.CO;2-2 10099596

[13] Carlsson P.-O. Palm F. Mattsson G., “Low revascularization of experimentally transplanted human pancreatic islets,” J. Clin. Endocrinol. Metab. 87, 5418–5423 (2002). 10.1210/jc.2002-020728 12466329

[r14] Nyqvist D. et al., “Donor islet endothelial cells participate in formation of functional vessels within pancreatic islet grafts,” Diabetes 54, 2287–2293 (2005). DIAEAZ 0012-1797 10.2337/diabetes.54.8.2287 16046293

[15] Toso C. et al., “Histologic graft assessment after clinical islet transplantation,” Transplantation 88, 1286–1293 (2009). TRPLAU 0041-1337 10.1097/TP.0b013e3181bc06b0 19996928

[16] Carrier R. L. et al., “Perfusion improves tissue architecture of engineered cardiac muscle,” Tissue Eng. 8, 175–188 (2002).1937-3341 10.1089/107632702753724950 12031108

[17] Nih L. R. et al., “Dual-function injectable angiogenic biomaterial for the repair of brain tissue following stroke,” Nat. Mater. 17, 642–651 (2018). NMAACR 1476-1122 10.1038/s41563-018-0083-8 29784996 PMC6019573

[18] Andreeva E. R. et al., “Response of adipose tissue-derived stromal cells in tissue-related microenvironment to short-term hypoxic stress,” Cells Tissues Organs 200, 307–315 (2014).1422-6405 10.1159/000438921 26407140

[19] Rosová I. et al., “Hypoxic preconditioning results in increased motility and improved therapeutic potential of human mesenchymal stem cells,” Stem Cells 26, 2173–2182 (2008). 10.1634/stemcells.2007-1104 18511601 PMC3017477

[20] Anders J. J. Lanzafame R. J. Arany P. R., “Low-level light/laser therapy versus photobiomodulation therapy,” Photomed. Laser Surg. 33, 183–184 (2015). 10.1089/pho.2015.9848 25844681 PMC4390214

[21] Baxter G. D., Therapeutic Lasers: Theory and Practice, p. 112, Churchill Livingstone, Edinburgh (1994).

[r22] Tuner J., Laser Therapy: Clinical Practice And Scientific Background: A Guide for Research Scientists, Doctors, Dentists, Veterinarians and Other Interested Parties within the Medical Field, Prima Books, Tallinn, Estonia (2002).

[r23] Chung H. et al., “The nuts and bolts of low-level laser (light) therapy,” Ann. Biomed. Eng. 40, 516–533 (2012). ABMECF 0090-6964 10.1007/s10439-011-0454-7 22045511 PMC3288797

[r24] Karu T. I., Ten Lectures on Basic Science of Laser Phototherapy, Prima Books, Coeymans Hollow, New York (2007).

[r25] Chailakhyan R. K. et al., “Activation of bone marrow multipotent stromal cells by laser and EHF radiation and their combined impacts,” Sovrem. Technol. Med. 9, 28 (2017). 10.17691/stm2017.9.1.03

[r26] Andreeva N. V. et al., “The effect of infrared laser irradiation on the growth of human melanoma cells in culture,” Biophysics 61, 979–984 (2016). BIOPAE 0006-3509 10.1134/S000635091606004X

[r27] Sanderson T. H. et al., “Inhibitory modulation of cytochrome c oxidase activity with specific near-infrared light wavelengths attenuates brain ischemia/reperfusion injury,” Sci. Rep. 8, 3481 (2018). SRCEC3 2045-2322 10.1038/s41598-018-21869-x 29472564 PMC5823933

[28] Zein R. Selting W. Hamblin M. R., “Review of light parameters and photobiomodulation efficacy: dive into complexity,” J. Biomed. Opt. 23, 120901 (2018). JBOPFO 1083-3668 10.1117/1.JBO.23.12.120901 PMC8355782 30550048

[29] Turrioni A. et al., “Transdentinal cell photobiomodulation using different wavelengths,” Oper. Dent. 40, 102–111 (2015). 10.2341/13-370-L 25136901

[30] Zhu W. et al., “3D printing scaffold coupled with low level light therapy for neural tissue regeneration,” Biofabrication 9, 025002 (2017). 10.1088/1758-5090/aa6999 28349897

[31] Theocharidou A. et al., “Odontogenic differentiation and biomineralization potential of dental pulp stem cells inside Mg-based bioceramic scaffolds under low-level laser treatment,” Lasers Med. Sci. 32, 201–210 (2017). 10.1007/s10103-016-2102-9 27785631

[32] de Vasconcellos L. M. R. et al., “Erratum to: titanium scaffold osteogenesis in healthy and osteoporotic rats is improved by the use of low-level laser therapy (GaAlAs) (Lasers Med Sci, (2016), 31, (899-905), 10.1007/s10103-016-1930-y),” Lasers Med. Sci. 32, 733 (2017). 10.1007/s10103-017-2167-0 27056701

[33] Fekrazad R. et al., “The effects of combined low level laser therapy and mesenchymal stem cells on bone regeneration in rabbit calvarial defects,” J. Photochem. Photobiol. B 151, 180–185 (2015). 10.1016/j.jphotobiol.2015.08.002 26298068

[34] Karu T. I., “Mitochondrial signaling in mammalian cells activated by red and near-IR radiation,” Photochem. Photobiol. 84, 1091–1099 (2008). PHCBAP 0031-8655 10.1111/j.1751-1097.2008.00394.x 18651871

[35] Fekrazad R. et al., “Photobiomodulation with single and combination laser wavelengths on bone marrow mesenchymal stem cells: proliferation and differentiation to bone or cartilage,” Lasers Med. Sci. 34, 115–126 (2019). 10.1007/s10103-018-2620-8 30264177 PMC6344244

[36] George S. Hamblin M. R. Abrahamse H., “Effect of red light and near infrared laser on the generation of reactive oxygen species in primary dermal fibroblasts,” J. Photochem. Photobiol. B 188, 60–68 (2018). JPPBEG 1011-1344 10.1016/j.jphotobiol.2018.09.004 30216761 PMC6214457

[37] Garrido P. R. et al., “Effects of photobiomodulation therapy on the extracellular matrix of human dental pulp cell sheets,” J. Photochem. Photobiol. B 194, 149–157 (2019). 10.1016/j.jphotobiol.2019.03.017 30954874

[r38] Diniz I. M. A. et al., “Photobiomodulation of mesenchymal stem cells encapsulated in an injectable rhBMP4-loaded hydrogel directs hard tissue bioengineering,” J. Cell. Physiol. 233, 4907–4918 (2018). JCLLAX 0021-9541 10.1002/jcp.26309 29215714

[39] Basso F. G. et al., “Low-level laser therapy in 3D cell culture model using gingival fibroblasts,” Lasers Med. Sci. 31, 973–978 (2016). 10.1007/s10103-016-1945-4 27126408

[40] Yang L. et al., “Photobiomodulation preconditioning prevents cognitive impairment in a neonatal rat model of hypoxia-ischemia,” J. Biophotonics 12, e201800359 (2019). 10.1002/jbio.201800359 30652418 PMC6546525

[41] Wachal K. et al., “Physical properties of hydrogel wound dressing and its use in low-level laser therapy (LLLT),” Lasers Med. Sci. 33, 1317–1325 (2018). 10.1007/s10103-018-2484-y 29611063

[42] Lukomska B. et al., “Challenges and controversies in human mesenchymal stem cell therapy,” Stem Cells Int. 2019, 1–10 (2019). 10.1155/2019/9628536 PMC6481040 31093291

[43] Mitrano T. I. et al., “Culture and characterization of mesenchymal stem cells from human gingival tissue,” J. Periodontol. 81, 917–925 (2010). 10.1902/jop.2010.090566 20450355

[44] Zhang Q. et al., “Mesenchymal stem cells derived from human gingiva are capable of immunomodulatory functions and ameliorate inflammation-related tissue destruction in experimental colitis,” J. Immunol. 183, 7787–7798 (2009). JOIMA3 0022-1767 10.4049/jimmunol.0902318 19923445 PMC2881945

[45] Jin S. H. et al., “Isolation and characterization of human mesenchymal stem cells from gingival connective tissue,” J. Periodontal Res. 50, 461–467 (2015). 10.1111/jre.12228 25229614

[46] Zhao J. et al., “A preclinical study—systemic evaluation of safety on mesenchymal stem cells derived from human gingiva tissue,” Stem Cell Res. Ther. 10, 165 (2019). 10.1186/s13287-019-1262-5 31196163 PMC6567625

[47] Zhang X. et al., “A protocol for isolation and culture of mesenchymal stem cells from human gingival tissue,” Am. J. Clin. Exp. Immunol. 8, 21–26 (2019). 10.1038/nprot.2009.238 31497379 PMC6726972

[48] Lee H. et al., “A study of the effects of doxorubicin-containing liposomes on osteogenesis of 3D stem cell spheroids derived from gingiva,” Materials 12, 2693 (2019). MATEG9 1996-1944 10.3390/ma12172693 PMC6747561 31443583

[49] Tomasello L. et al., “Mesenchymal stem cells derived from inflamed dental pulpal and gingival tissue: a potential application for bone formation,” Stem Cell Res. Ther. 8, 179 (2017). 10.1186/s13287-017-0633-z 28764802 PMC5540218

[50] Zorin V. L. et al., “Myogenic potential of human alveolar mucosa derived cells,” Cell Cycle 16, 545–555 (2017).1538-4101 10.1080/15384101.2017.1284714 28118065 PMC5384593

[51] Ansari S. et al., “Muscle tissue engineering using gingival mesenchymal stem cells encapsulated in alginate hydrogels containing multiple growth factors,” Ann. Biomed. Eng. 44, 1908–1920 (2016). ABMECF 0090-6964 10.1007/s10439-016-1594-6 27009085 PMC4880526

[52] Gao Y. et al., “Isolation and multiple differentiation potential assessment of human gingival mesenchymal stem cells,” Int. J. Mol. Sci. 15, 20982–20996 (2014).1422-0067 10.3390/ijms151120982 25405732 PMC4264207

[53] Kober J. et al., “Generation of a fibrin based three-layered skin substitute,” Biomed Res. Int. 2015, 1–8 (2015). 10.1155/2015/170427 PMC4508374 26236715

[r54] Park J. S. et al., “Chondrogenesis of human mesenchymal stem cells in fibrin constructs evaluated *in vitro* and in nude mouse and rabbit defects models,” Biomaterials 32, 1495–1507 (2011). BIMADU 0142-9612 10.1016/j.biomaterials.2010.11.003 21122912

[55] Shpichka A. I. et al., “Evaluation of the vasculogenic potential of hydrogels based on modified fibrin,” Cell Tissue Biol. 11, 81–87 (2017). 10.1134/S1990519X17010126

[56] Shpichka A. I. et al., “Digging deeper: structural background of PEGylated fibrin gels in cell migration and lumenogenesis,” RSC Adv. 10, 4190–4200 (2020). 10.1039/C9RA08169K PMC9049040 35495227

[57] Lalwani G. et al., “Porous three-dimensional carbon nanotube scaffolds for tissue engineering,” J. Biomed. Mater. Res. A 103, 3212–3225 (2015). 10.1002/jbm.a.35449 25788440 PMC4552611

[58] Sproul E. P. Hannan R. T. Brown A. C., “Controlling fibrin network morphology, polymerization, and degradation dynamics in fibrin gels for promoting tissue repair,” in Biomaterials for Tissue Engineering: Methods and Protocols, Chawla K., Ed., Vol. 1758, pp. 85–99, Humana Press Inc., New York (2018). 10.1007/978-1-4939-7741-3_7 PMC12989979 29679324

[59] Efremov Y. M. et al., “Measuring nanoscale viscoelastic parameters of cells directly from AFM force-displacement curves,” Sci. Rep. 7, 1541 (2017). SRCEC3 2045-2322 10.1038/s41598-017-01784-3 28484282 PMC5431511

[60] Dominici M. et al., “Minimal criteria for defining multipotent mesenchymal stromal cells. The International Society for Cellular Therapy position statement,” Cytotherapy 8, 315–317 (2006). 10.1080/14653240600855905 16923606

[61] Zahm J. M. et al., “Chronology of cellular alterations during 7-ketocholesterol-induced cell death on A7R5 rat smooth muscle cells: analysis by time lapse-video microscopy and conventional fluorescence microscopy,” Cytom. Part A 52, 57–69 (2003).1552-4922 10.1002/cyto.a.10027 12655649

[r62] Cselenyák A. et al., “Live-cell fluorescent imaging of membrane or mitochondrion transfer between connected cells in culture,” in Microscopy Science: Technology, Application and Education, Mendez-Vilas A. Dõaz J., Eds., Vol. 1, pp. 764–771, Formatex, Badajoz, Spain (2010).

[r63] Gonzalez S. et al., “*In vivo* time-lapse imaging of mitochondria in healthy and diseased peripheral myelin sheath,” Mitochondrion 23, 32–41 (2015). 10.1016/j.mito.2015.05.004 26031781

[r64] Duval K. et al., “Modeling physiological events in 2D vs. 3D cell culture,” Physiology 32, 266–277 (2017). 10.1152/physiol.00036.2016 28615311 PMC5545611

[65] Chazotte B., “Labeling mitochondria with mitotracker dyes,” Cold Spring Harb. Protoc. 2011, 990–992 (2011). 10.1101/pdb.prot5648 21807856

[66] Reiss R. F. Oz M. C., “Autologous fibrin glue: production and clinical use,” Trans. Med. Rev. 10, 85–92 (1996). TMEREU 0887-7963 10.1016/S0887-7963(96)80085-X 8721966

[r67] Hartman A. R., “Autologous whole plasma fibrin gel,” Arch. Surg. 127, 357 (1992). 10.1001/archsurg.1992.01420030135026 1550487

[68] Kumar V. Chapman J. R., “Autologous thrombin: intraoperative production from whole blood,” J. Extra-Corpor. Technol. 40, 94–98 (2008). 18705544 PMC4680638

[69] Perumcherry S. R. et al., “A novel method for the fabrication of fibrin-based electrospun nanofibrous scaffold for tissue-engineering applications,” Tissue Eng. Part C 17, 1121–1130 (2011). 10.1089/ten.tec.2010.0734 21902615

[70] Yao S. et al., “Hierarchically aligned fibrin nanofiber hydrogel accelerated axonal regrowth and locomotor function recovery in rat spinal cord injury,” Int. J. Nanomed. 13, 2883–2895 (2018). 10.2147/IJN.S159356 PMC5961640 29844671

[71] Wozniak G., “Fibrin sealants in supporting surgical techniques: the importance of individual components,” Cardiovasc. Surg. 11, 17–21 (2003). 10.1016/S0967-2109(03)00067-X 12869984

[72] Mol A. et al., “Fibrin as a cell carrier in cardiovascular tissue engineering applications,” Biomaterials 26, 3113–3121 (2005). BIMADU 0142-9612 10.1016/j.biomaterials.2004.08.007 15603806

[73] Koroleva A. et al., “Hydrogel-based microfluidics for vascular tissue engineering,” BioNanoMaterials 17, 19–32 (2016). 10.1515/bnm-2015-0026

[74] Sviridov A. P. et al., “Optical fields in porous polylactide matrices,” Quantum Electron. 50, 81–86 (2020). QUELEZ 1063-7818 10.1070/QEL17236

[75] Hu Y. et al., “Using indentation to characterize the poroelasticity of gels,” Appl. Phys. Lett. 96, 121904 (2010). APPLAB 0003-6951 10.1063/1.3370354

[76] Yusupov V. I. et al., “Optical properties of porous polylactide scaffolds,” Proc. SPIE 10716, 107161U (2018). PSISDG 0277-786X 10.1117/12.2315006

[77] Hadis M. A. et al., “The dark art of light measurement: accurate radiometry for low-level light therapy,” Lasers Med. Sci. 31, 789–809 (2016). 10.1007/s10103-016-1914-y 26964800 PMC4851696

[78] Abrahamse H. et al., “Fluence and wavelength of low intensity laser irradiation affect activity and proliferation of human adipose derived stem cells,” Med. Technol. SA 24, 15–20 (2010).

[79] Winter R. et al., “Photobiomodulation (PBM) promotes angiogenesis *in-vitro* and in chick embryo chorioallantoic membrane model,” Sci. Rep. 8, 17080 (2018). SRCEC3 2045-2322 10.1038/s41598-018-35474-5 30459437 PMC6244005

[80] Shanmugapriya K. et al., “Multifunctional heteropolysaccharide hydrogel under photobiomodulation for accelerated wound regeneration,” Ceram. Int. 46, 7268–7278 (2020). 10.1016/j.ceramint.2019.11.221

[81] Bikmulina P. Y. et al., “Photobiomodulation enhances mitochondrial respiration in an *in vitro* rotenone model of Parkinson’s disease,” Opt. Eng. 59, 061620 (2020). 10.1117/1.OE.59.6.061620

[82] Zhu T. et al., “Irradiation by blue light-emitting diode enhances osteogenic differentiation in gingival mesenchymal stem cells *in vitro*,” Lasers Med. Sci. 34, 1473–1481 (2019). 10.1007/s10103-019-02750-3 30826951

[83] Karu T. I., “Molecular mechanisms of the therapeutic effect of low-intensity laser radiation,” Lasers Life Sci. 2, 53–74 (1988).

[84] Wang Y. et al., “Red (660 nm) or near-infrared (810 nm) photobiomodulation stimulates, while blue (415 nm), green (540 nm) light inhibits proliferation in human adipose-derived stem cells,” Sci. Rep. 7, 7781 (2017). SRCEC3 2045-2322 10.1038/s41598-017-07525-w 28798481 PMC5552860

[85] Sturgill T. W. et al., “Insulin-stimulated MAP-2 kinase phosphorylates and activates ribosomal protein S6 Kinase II,” Nature 334, 715–718 (1988). 10.1038/334715a0 2842685

[86] Zhang J. Xing D. Gao X., “Low-power laser irradiation activates Src tyrosine kinase through reactive oxygen species-mediated signaling pathway,” J. Cell. Physiol. 217, 518–528 (2008). JCLLAX 0021-9541 10.1002/jcp.21529 18615581

[87] Teuschl A. et al., “Phototherapy with LED light modulates healing processes in an *in vitro* scratch-wound model using 3 different cell types,” Dermatol. Surg. 41, 261–268 (2015). 10.1097/DSS.0000000000000266 25654197

